# Advanced Aerogels for Water Remediation: Unraveling Their Potential in Fats, Oils, and Grease Sorption—A Comprehensive Review

**DOI:** 10.3390/gels11040268

**Published:** 2025-04-04

**Authors:** Adina-Elena Segneanu, Dumitru-Daniel Herea, Gabriela Buema, Ionela Amalia Bradu, Melinda Cepan, Ioan Grozescu

**Affiliations:** 1 Department of Chemistry, Institute for Advanced Environmental Research, West University of Timişoara (ICAM–WUT), Oituz Street, 300086 Timişoara, Romania; adina.segneanu@e-uvt.ro (A.-E.S.); ionela.potinteu@e-uvt.ro (I.A.B.); 2 National Institute of Research and Development for Technical Physics, 15 Dimitrie Mangeron Avenue, 700050 Iaşi, Romania; 3 Department of Applied Chemistry and Engineering of Inorganic Compounds and the Environment, University Politehnica Timisoara, 2 Piata Victoriei, 300006 Timişoara, Romania; cepan.melinda@gmail.com (M.C.); ioangrozescu@gmail.com (I.G.)

**Keywords:** aerogel materials, water remediation, oil sorption, organic solvents sorption, oil/water separation

## Abstract

The increasing contamination of water bodies by fats, oils, and grease (FOG) poses significant environmental and operational challenges, necessitating the development of advanced remediation technologies. Aerogels, with their ultra-lightweight structure, high porosity, and tunable surface chemistry, have emerged as promising sorbents for efficient FOG removal. This comprehensive review explores recent advancements in aerogel materials, highlighting novel formulations, functional modifications, and nanotechnology integrations that enhance sorption capacity and reusability. It delves into the mechanistic aspects of FOG sorption, providing insights into how surface interactions and structural properties influence performance. The sustainability of aerogels is emphasized, particularly the use of bio-based and eco-friendly materials that align with green remediation strategies. A comparative analysis with conventional sorbents underscores the advantages of aerogels in terms of efficiency, environmental impact, and cost-effectiveness. Furthermore, real-world applications, including oil spill cleanup and wastewater treatment, are discussed alongside challenges, regulatory considerations, and future research directions. By offering a holistic perspective on the potential of aerogels in water remediation, this review serves as a valuable resource for researchers and industry professionals seeking innovative and sustainable solutions for FOG management.

## 1. Introduction

Polluted wastewater is a critical global challenge exacerbated by rapid industrialization, technological advancements, and economic growth. Left untreated, wastewater poses serious environmental risks and threatens public health [1]. Both industrial and domestic wastewater serve as major sources of pollution, introducing a wide range of contaminants that disrupt ecosystems. These pollutants contribute to eutrophication, aquatic toxicity, and disturbances in terrestrial environments, ultimately endangering public well-being [2].

Among the myriad contaminants found in wastewater, fats, oils, and greases (FOGs) pose a particularly intricate challenge due to their distinctive physical properties and considerable environmental repercussions [3]. FOGs predominantly enter wastewater systems from both domestic and industrial sources, such as restaurants and food processing facilities. Over time, these substances accumulate, leading to serious blockages in pipes, diminishing the efficacy of biological treatment processes, and contaminating natural water bodies—ultimately degrading water quality and endangering aquatic ecosystems [4,5,6]. Traditional methods of FOG removal, including degreasing, chemical treatments, and biological processes, often face significant hurdles, including high operational costs, incomplete contaminant removal, and the risk of secondary pollution. These challenges have catalyzed the quest for innovative, efficient, and sustainable technologies. Among the promising solutions being explored is the use of aerogels [7,8].

Aerogels, characterized by their micro- and nanoscale porous structures, have emerged as extraordinary materials for pollutant sorption. The term “aerogel” describes a gel-like substance in which liquid molecules are replaced by air, yielding an ultra-light, highly porous solid. These remarkable materials are gaining traction across various fields, including wastewater treatment, drug delivery, the food industry, and medical devices [9,10,11,12]. Even more, different types of aerogels were reported for tissue regeneration, wound healing, and diagnostics or as antibacterial agents [13,14,15]. However, in spite of their extensive application range, the known brittleness of aerogels makes their processing and handling difficult. Consequently, a series of conventional approaches have been considered to improve their mechanical durability, such as hydrolytic polycondensation and addition condensation, epoxide-assisted gelation, nucleation and crystal growth, or carbonization, and new methods, such as self-assembly processes, hydrothermal, click chemistry, and double or multi-cross-linking [16]. The main characteristics that recommend them for use in wastewater treatment are their low cost and the fact that they are durable, versatile, and environmentally friendly [17,18,19]. Aerogels can be synthesized from natural, synthetic, or hybrid materials, which can then be loaded with various functional particles, or their surface can be chemically modified to increase efficiency depending on the application [19]. Aerogels are the lightest materials known today, consisting of 90–99% air. They can be adjusted in terms of their hydrophobic or hydrophilic properties and are very easy to recycle [17].

Over the years, various types of aerogels have been developed for water purification. The most commonly studied variants encompass polymer-based aerogels, such as chitosan, cellulose, graphene oxide, and silica dioxide. The earliest aerogels, synthesized in the 1970s, were primarily silica-based [20,21,22]. As research progressed, carbon-based aerogels emerged, followed by graphene-based aerogels introduced in 2010, marking a significant advancement in material science [23,24].

This review represents a groundbreaking contribution to the field of water remediation, highlighting the remarkable potential of advanced aerogels as highly efficient sorbents for fats, oils, and grease (FOG). By examining state-of-the-art material innovations, including novel hybrid formulations and functionalized aerogels, the review illustrates how these advancements significantly enhance sorption capacities. Central to this discussion are new insights into the mechanisms behind FOG sorption, with a particular focus on the critical roles of surface chemistry and structural properties in optimizing performance. The review is imbued with a strong emphasis on sustainability, highlighting the development of bio-based aerogels that are in harmony with eco-friendly remediation practices. Through a thorough comparative analysis of traditional sorbents versus advanced aerogels, this review convincingly demonstrates the superior efficiency, reusability, and reduced environmental impact of aerogels. It also delves into innovative real-world applications, such as oil spill cleanup and wastewater treatment, substantiated by compelling case studies and experimental data that underscore the practical relevance of these materials. Moreover, the review explores the integration of nanotechnology in aerogel design, detailing how nanoscale modifications can dramatically enhance sorption properties. The discussion extends to the multi-functionality of aerogels, revealing their potential roles in catalysis and pollutant degradation, thus broadening their applicability in environmental remediation.

Future research directions and in-depth mechanistic studies are proposed, offering a pathway for a deeper understanding of aerogel performance. Additionally, the review addresses economic and regulatory considerations, providing a comprehensive outlook on the practical implementation of these innovative materials.

In summary, this review is not only a comprehensive resource but also a catalyst for innovation and progress in sustainable water remediation technologies. It invites researchers, practitioners, and policymakers to engage with these cutting-edge developments, positioning advanced aerogels as a pivotal solution in the quest for effective and environmentally responsible water treatment methods.

## 2. Aerogels as Sorption Materials for Oils/Organic Solvents

### 2.1. Background

Water polluted with oils is a cause for concern, apart from water contamination with heavy metals, dyes, or antibiotics. Li and collaborators [25] highlighted in their publication review that the negative impacts on the environment and human social development are caused by the frequent oil spills and oily wastewater generated by everyday life from various sources [25].

Oil/water mixtures typically fall into two categories: (i) those that exist in a homogeneous phase and (ii) those that display a distinct separation between the water and oil phases [25]. This classification is critical for devising appropriate remedial strategies.

Grease (oil) pollution of industrial or municipal water sources is multifaceted and primarily originates from petroleum products used in the oil industry to obtain gasoline and diesel, steelmaking and metalworking, car services, vegetable and animal oil derivatives, or waste resulting from food processing. The nature of these fats (oils) is of the type of light and heavy hydrocarbons, oils for lubricating machine parts or in cutting processes, not emulsified or found in plants and animals [25]. The nature of these oils can vary widely, encompassing both light and heavy hydrocarbons, as well as lubricating oils used for machinery and cutting processes. Many of these pollutants are often not emulsified, existing instead in their pure form, which can complicate remediation efforts. Understanding the characteristics and behaviors of these oil types is essential for developing effective sorption materials, like aerogels, which can be engineered to selectively adsorb these contaminants and mitigate their impact on our water resources [25].

A series of techniques, methods, and technologies are used to treat wastewater with oils and fats. These include wastewater treatment by chemical processes, separation in a gravitational field, and biological sorption processes on various types of materials with adsorbent properties. In the case of oils in the form of droplets with dimensions of the order of micrometers, separation techniques with absorbent membranes or dissolved air flotation devices are used. These techniques are, however, expensive and involve high-pressure processes. A method that has been proven extremely effective in treating water polluted with fats (oils) is sorption on materials with high porosity and specific surface area.

To address oil contamination in wastewater, several treatment techniques have been developed, including chemical treatment, gravitational separation, biological degradation, and sorption processes utilizing various adsorbent materials. When oil is dispersed in water as microdroplets, advanced separation techniques such as absorbent membranes and dissolved air flotation devices are required. However, these methods often involve high operational costs and energy-intensive processes. Among the most effective approaches for oil removal is sorption using materials with high porosity and a large specific surface area.

Sorption is widely recognized as an efficient method for oil removal from water, utilizing specialized sorbent materials [26,27,28]. One of the key characteristics that determine the efficiency of oil-absorbing materials is hydrophobicity, which defines their ability to repel water while selectively absorbing oil [29,30]. The water contact angle (WCA) measurement, denoted as θ, is commonly used to assess surface wettability properties. Surfaces with θ < 90° are classified as hydrophilic, while those with θ > 90° are considered hydrophobic. When θ exceeds 150°, the surface exhibits superhydrophobic properties, making it highly effective for oil separation applications [31]. Other critical parameters in evaluating oil-absorbing materials include the oil/solvent sorption capacity and oil/water separation efficiency, both of which can be determined through specific equations [32,33]. Additionally, the reusability of sorption materials is a key factor for assessing their practical application and long-term cost-effectiveness [34,35,36].

The equations for oil/solvent sorption capacity (Q, mg/g) and separation efficiency (η, %) are detailed below as Equations (1) and (2).

Equation/meaning of parameters

(1) $$Q=\frac{\left(m_{1}-m_{0}\right)}{m_{0}}$$

Q is oil/solvent sorption capacity (mg/g);

$m_{0}$ is the weight of aerogel before sorption (g); 

$m_{1}$ is the weight of aerogel after the sorption (g);

(2) $$\eta =\frac{m_{a}}{m_{b}}\times 100$$

η is the separation efficiency (%);

$m_{a}$ is the volume of oil after filtration; 

$m_{b}$ is the initial volume of the oil/water mixture.

### 2.2. Aerogels: Advanced Materials for Oil Removal

Aerogels have emerged as highly efficient absorbents for treating oily wastewater due to their ultra-high porosity, tunable pore structure, large surface area, high sorption capacity, and lightweight nature [23,37,38,39]. Our previously published review [40] provides a comprehensive overview of aerogel classification, preparation methods, characterization techniques, and properties.

Aerogels can be synthesized from a variety of materials, including activated carbon, wood-processing waste, clay, zeolites, and hydrophobic plant-derived compounds. While activated carbon is widely used for sorption applications, it suffers from limitations such as slow sorption kinetics and restricted pollutant-loading capacity [27]. Therefore, research is focused on developing new materials with low water affinity and high sorption efficiency to improve oil removal performance.

#### 2.2.1. Synthesis Routes for Hydrophobic and Superoleophilic Aerogels

The sol-gel method is one of the most effective techniques for synthesizing aerogels with high hydrophobicity, superoleophilicity, and excellent oil sorption capacity, making them ideal for wastewater treatment [41]. One such aerogel, featuring a sponge-like structure, was synthesized using the sol-gel process followed by supercritical drying. The synthesis involved methyltriethoxysilane (MTES) and dimethyldiethoxysilane (DMDEOS) as precursors, ethanol as the solvent, hexadecyltrimethylammonium bromide as the surfactant, and hydrochloric acid and ammonia as catalysts [41].

The procedure was carried out in three stages:

(i) *Hydrolysis:*

A homogeneous solution was prepared by dissolving the surfactant (hexadecyltrimethylammonium) in an ethanol/water mixture, followed by the addition of precursors (MTES, DMDEOS) and hydrochloric acid [41].

(ii) *Condensation and gelation:*

Ammonia was added to promote condensation and the mixture was transferred into sealed containers for gelation at ambient temperature. The gel was then matured in ethanol for 72 h [41].

(iii) *Supercritical drying:*

The aerogel was dried under supercritical conditions at 260 °C and 10 MPa, resulting in a highly porous structure optimized for oil sorption [41].

Another type of aerogel with very-low-density and high hydrophobicity was synthesized using silsesquioxane dried under vacuum in the presence of precursor monomers terephthalaldehyde and 3-aminopropyl-triethoxysilane, with acetic acid as a catalyst [42]. The procedure involved mixing terephthalaldehyde/3-aminopropyl-triethoxysilane with methyltrimethoxysilane in an ethanol solution, followed by ultrasonication. A small amount of water and acetic acid was then added, and the mixture was ultrasonicated again before being transferred to a sealed cylindrical container and heated at 60 °C for 12 h [42]. The resulting gel was washed multiple times with ethanol and subsequently dried at 40 °C and 80 °C for 12 h [42]. The presence of the catalyst significantly influenced the gelation mechanism, leading to monolithic aerogels with enhanced porosity and low density, making them highly suitable for oil/water separation [42].

#### 2.2.2. Silica-Based Aerogels

Silica-based aerogels have emerged as a highly promising material for oil sorption due to their ultra-low density, high porosity, and exceptional specific surface area [26]. These aerogels are typically synthesized via the sol-gel method, forming nanometric granules with superior sorption properties [26]. The primary precursor used is tetramethoxysilane (TMOS), and to enhance hydrophobicity, the Si-OH functional groups are chemically modified with Si-CH_3_, C_2_H_5_, or CF_3_(CH_2_)_2_ groups. This functionalization allows silica aerogels to efficiently adsorb oils and organic pollutants, whether they are miscible or immiscible in water [26].

Silica aerogels can be categorized into two main types:

(a) Hydrophobic silica aerogels are ideal for adsorbing non-polar organic compounds that have low water solubility, such as oils and hydrocarbons [26];
(b) Hydrophilic silica aerogels are more effective for water-soluble organic pollutants, making them useful in broader wastewater treatment applications [26].

A highly effective technique for utilizing silica aerogels in oil/water separation is reverse fluidization, where a water/oil emulsion flows downward through an aerogel nanoparticle bed. Studies indicate that aerogel nanoparticles of varying sizes can effectively separate oil from water, with sorption efficiency influenced by:

(i) Nanoparticle size and density in solution;
(ii) Thickness of the oil layer;
(iii) Flow rate and volume of the contaminated fluid.

Under optimal conditions, silica aerogel nanoparticles can adsorb oil up to 2.8 times their weight, making them a highly efficient solution for oil spill cleanup and wastewater treatment [26].

#### 2.2.3. Aerogels from Natural Polysaccharides

Natural polysaccharide-based aerogels are gaining significant attention as a sustainable and eco-friendly alternative for oil removal from water. Derived from renewable sources such as agricultural waste, plant biomass, and cellulose, these aerogels offer a cost-effective solution for extracting oily pollutants, fats, and other hazardous contaminants that pose risks to aquatic ecosystems and human health [26].

One of the most widely used polysaccharides for aerogel synthesis is cellulose, commonly sourced from agricultural residues [26].

Cellulose-based aerogels possess several key advantages over conventional oil-absorbing materials, including the following:

(i) Ultra-lightweight nature, significantly lighter than water;
(ii) Extremely low density, allowing for efficient flotation and sorption;
(iii) Exceptionally high specific surface area, enhancing their oil-absorbing capacity [26].

Due to their biodegradability, renewability, and scalability, polysaccharide-based aerogels represent a highly promising material for wastewater treatment and environmental remediation. Their ability to selectively absorb oils while remaining stable in aqueous environments makes them an excellent candidate for large-scale oil spill recovery and industrial wastewater purification [26].

##### Cellulose-Based Aerogels

Cellulose is the primary structural component in the production of plant-derived aerogels, found abundantly in agricultural and food plants as well as industrial waste such as sawdust, paper, and cotton [43].

The sorption efficiency of cellulose aerogels for oil removal from wastewater is influenced by several factors, including pH levels, contact time, temperature, and initial oil concentration. The purification process, which is crucial for achieving high-quality aerogels, can be carried out through physical, chemical, or biological methods [43].

The production of cellulose aerogels involves multiple stages, with the formation of a porous 3D structure and drying being the most critical. The presence of nanometer-scale cellulose fibers significantly increases the specific surface area of the aerogel, enhancing its sorption efficiency. The synthesis process commonly includes vacuum lyophilization, which removes the solvent while preserving the aerogel’s structural network [43]. Additionally, supercritical drying using CO_2_ at controlled pressure and temperature is used to achieve highly porous aerogels. By gradually reducing pressure and temperature, the solvent transitions to a gaseous state, leaving behind a solid aerogel with ultra-low density and high porosity [43]. To further enhance sorption capabilities, chemical functionalization with amine, carboxyl, or magnetic groups can significantly improve oil/water separation efficiency [43]. The effectiveness of oil sorption also depends on the oil’s density, with low-density oils being more easily adsorbed [43].

Supercritical Drying and Structural Characteristics

According to Liu et al. [44], cellulose-based aerogels are primarily synthesized through supercritical drying, a method that produces aerogels with:

(a) High specific surface areas and porosities (≥90%);
(b) Mesoporous structures with pore sizes ranging between 2 and 50 nm [44].

Cellulose itself exhibits high crystallinity and strong intramolecular hydrogen bonding, making it difficult to dissolve in standard solvents. While derivatized solvents are recommended for solubilization, non-derivatized solvents are generally avoided due to their toxicity and poor recovery efficiency [44]. These aerogels are particularly valuable for oil sorption due to their biodegradability, renewability, and high BET surface area [45,46]. The structure and sorption efficiency of cellulose aerogels can be fine-tuned by selecting an appropriate cellulose source and preparation technique.

One of the processes for obtaining aerogels takes place in two stages and involves processing the gel from solutions or dispersions of precursors followed by drying it in air after removing the solvents [45,46]. A chemical method for processing aerogels, the sol-gel method, is a widely used chemical process for aerogel synthesis and offers several advantages, including the following:

(i) Low processing temperatures;
(ii) Minimal secondary reactions;
(iii) High material purity [45,46].

Drying the cellulose aerogel in the final stage is a very important operation to obtain a material with high performance. Two primary drying techniques are used in aerogel fabrication:

(i) Supercritical drying in CO_2_ preserves the aerogel’s porous structure but is costly and time-intensive [45,46];
(ii) Vacuum lyophilization uses sublimation to remove liquid and is preferred for large-scale, cost-effective production.

Supercritical drying is reserved for high-performance applications, while lyophilization is recommended for general-purpose aerogels due to its lower cost and shorter processing time [45,46].

##### Chemical Modifications for Enhanced Sorption

Liao et al. [47] synthesized a hydrophobic cellulose aerogel via chemical vapor deposition (CVD) using methyltrichlorosilane (MTMS). The modified aerogel demonstrated significantly improved sorption capacities compared to unmodified cellulose aerogels for the following:

(i) Pump oil: 59.32 g/g;
(ii) Colza oil: 55.85 g/g;
(iii) Chloroform: 46.23 g/g;
(iv) Methylbenzene: 40.16 g/g;
(v) Ethyl acetate, cyclohexane, n-hexane, petroleum ether: ~30 g/g [47].

In contrast, unmodified cellulose aerogels exhibited sorption capacities below 15 g/g for all tested pollutants [47]. The reusability study confirmed that the MTMS-modified aerogel retained high efficiency over 10 sorption cycles, with only slight decreases in sorption capacity for chloroform (from 1887 mg to 1591.9 mg) and pump oil (from 2106.7 mg to 1379.7 mg). Furthermore, colza oil, cyclohexane, and chloroform were completely absorbed within 2 s using the MTMS-modified cellulose aerogel [47].

##### Oil and Solvent Sorption Studies

Zhang et al. [48] investigated the oil and organic solvent sorption performance of cellulose-based aerogels. Various oils and solvents were tested, including gasoline, diesel, pump oil, corn oil, mineral oil, motor oil, acetone, ethanol, toluene, hexane, chloroform, and DMSO. The values of sorption capacities obtained are presented in Table 1 [48].

The modified aerogel maintained a sorption capacity higher than 84% even after 35 sorption–squeezing cycles. Additionally, the aerogel rapidly removed oils and solvents from gasoline/water and chloroform/water mixtures within minutes [48].

Zhang et al. [49] developed a novel 3D hierarchical nanocellulose aerogel foam (3D NAF/SDS) and explored the effects of varying concentrations of nanocellulose and sodium dodecyl sulfate (SDS) on the aerogel’s fabrication process. The study identified optimal concentrations of 0.4 wt% nanocellulose and 0.2 wt% SDS, which resulted in exceptional sorption capacities for the following solvents: (a) cyclohexane: 206.79 g/g and (b) ethyl acetate: 194.75 g/g [49].

##### Advanced Cellulose-Based Aerogels for High-Performance Sorption

**Figure 1:**
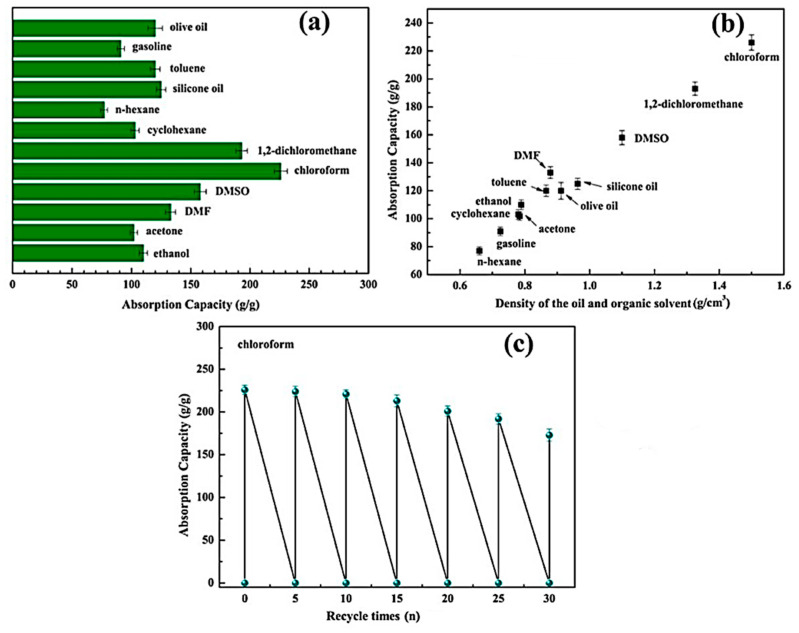
Sorption capacities of Si-CNF/BTCA aerogel for various organic liquids (**a**); The correlation of the sorption capacity of the Si-CNF/BTCA aerogel and the density of adsorbed oil and organic solvents (**b**); Recyclability of Si-CNF/BTCA aerogel for the sorption of chloroform within 30 cycles (**c**). Source [50].

Shang et al. [50] developed a highly porous nanocellulose aerogel (abbreviated Si-CNF/BTCA), which boasts an impressive porosity of 99.61% and a contact angle of 151° [46]. This aerogel was synthesized using a chemical vapor deposition (CVD) method involving hexadecyltrimethoxysilane (HTMS) [50]. The authors investigated the selective sorption capabilities of the Si-CNF/BTCA aerogel for a variety of oils and organic solvents, including olive oil, gasoline, toluene, silicone oil, n-hexane, cyclohexane, 1,2-dichloromethane, chloroform, dimethyl sulfoxide (DMSO), dimethyl formamide (DMF), acetone, and ethanol [50]. The sorption capacities ranged from 77 to 226 g/g, with the results illustrated in Figure 1a [50]. Additionally, Figure 1b presents the correlation between the sorption capacity of the Si-CNF/BTCA aerogel and the density of the adsorbed oils and solvents. The reusability study, depicted in Figure 1c, revealed that the Si-CNF/BTCA aerogel retained a high sorption capacity of 170 g/g for chloroform even after 30 cycles [50].

Hu and colleagues [51] developed a novel bacterial cellulose aerogel for efficient oil/water separation by incorporating methyltrimethoxysilane (MTMS) into a sulfonated nano-fibrillated bacterial cellulose (SNBC) matrix using a freeze-drying (lyophilization) method. Lyophilization is a method based on the sublimation of the template solvent after its solidification and is typically used to synthesize porous structures [52]. The resulting aerogels were designated as HBCA-X, where X represents the MTMS content. The materials were evaluated for their sorption capabilities with various solvents, including DMF (N,N-dimethylformamide), n-hexane, ethyl alcohol, silicone oil, and DCM (dichloromethane) [51]. The findings revealed that all synthesized aerogels exhibited impressive sorption capacities, ranging from 12.46 to 102.24 g/g. Notably, the HBCA-2 aerogel, containing 2% MTMS, demonstrated exceptional hydrophobicity with a contact angle of 152.4° and an oil sorption capacity between 42.14 g/g and 85.37 g/g [51]. Furthermore, the HBCA-2 material was subjected to cyclic sorption tests with silicone oil, retaining approximately 90% of its initial sorption capacity after 10 cycles. In continuous oil/water separation processes, HBCA-2 achieved a remarkable separation efficiency of 98.48%. Overall, the authors concluded that this engineered aerogel holds considerable promise for addressing large-scale environmental remediation associated with oil contamination, particularly for oil spill cleanup and wastewater treatment [51].

Cellulose-based aerogels are proving to be highly efficient, environmentally friendly, and scalable materials for oil sorption and wastewater purification. Their performance can be enhanced through chemical modifications, structural optimization, and tailored synthesis techniques [53]. The continued development of superhydrophobic and highly porous aerogels presents a viable pathway for tackling global challenges related to oil contamination and industrial wastewater treatment [53].

### 2.3. Reuse of Waste as Cellulose Sources for New Aerogels

#### 2.3.1. Paper Waste as a Cellulose Source

Paper waste serves as a valuable source of cellulose, aligning with circular economy principles by promoting material recovery and recycling. Utilizing this waste contributes significantly to forest conservation and pollution reduction. Through a series of separation processes, cellulose fibers are extracted and dispersed in water with kymene, then subjected to sonication, freezing, and gelation. The gel undergoes lyophilization and freeze-drying before being cured at 120 °C to cross-link kymene molecules [54]. Following lyophilization, the gel is coated with methyltrimethoxysilane via the chemical vapor deposition (CVD) technique, yielding aerogels with ultra-high porosity and extreme hydrophobicity [54]. These characteristics make them highly effective in oil sorption applications, particularly in oil spill remediation. Studies indicate that kymene-based cross-linkers outperform sodium hydroxide and urea, achieving a sorption efficiency of 95 g/g with an aerogel concentration of just 0.25% by weight. This formulation results in the lowest density and highest porosity (99.4%) [54]. Additionally, carbon aerogels synthesized from waste paper undergo oxidation, drying, and carbonization processes. These aerogels exhibit superhydophilicity, ultra-lightweight properties, and exceptional oil sorption capacity for water purification [55].

The freeze-drying process of cellulose nanofibers, derived from the chemical vapor deposition (CVD) of hexadecyltrimethoxysilane at varying weight concentrations, results in aerogels that exhibit remarkable oil sorption capabilities from aqueous environments. These modified aerogels are characterized by their exceptional hydrophobicity, lightweight structure, and enhanced sorption capacity [55].

To produce the modified aerogel, cellulose nanofibers were first diluted and uniformly dispersed in deionized water. This mixture was subjected to gentle stirring at ambient temperature to ensure even distribution of the nanofibers [55]. Following this, the solution was rapidly frozen using liquid nitrogen, a step crucial for preserving the structural integrity of the nanofibers [55]. The frozen material was then subjected to freeze-drying under low pressure, effectively removing moisture while maintaining the porous architecture of the aerogel [55]. The resulting aerogels not only demonstrate superior oil sorption properties but also retain the lightweight nature and hydrophobic characteristics necessary for various applications, including environmental remediation and industrial oil spill clean-up [55].

In another study, Thai and colleagues [56] synthesized a range of cellulose-based aerogels derived from sugarcane bagasse specifically designed for oil spill remediation. The oil sorption capacity of these innovative materials was evaluated using crude oil as a test medium [56]. Remarkably, the results demonstrated that the aerogel could absorb oil up to 25 times its initial weight, highlighting its exceptional efficacy in addressing environmental challenges associated with oil spills [56]. This significant finding underscores the potential of cellulose-based aerogels as sustainable solutions for ecological restoration [56].

#### 2.3.2. Alternative Cellulose Sources for Aerogel Synthesis

The cellulose derived from chestnut shell fibers served as the precursor for synthesizing hydrophobic bio-aerogels [33]. The synthesized aerogels, labeled as x% y: z (where x represents cellulose concentration and y: z denotes the ratio of cellulose to BTMSE [1,2-bis(trimethoxysilyl)ethane]), were evaluated for the following:

(i) Oil Sorption Capacity: This assessment involved immersing the aerogel, previously weighed, in an oil or organic solvent for 3 min to allow for equilibrium. Afterward, the aerogel was retrieved and reweighed to determine the amount of oil absorbed [33].
(ii) Oil/Water Separation: A high-speed swirling technique was employed for 2 h to create an emulsion of an incompatible oil/water mixture (50/50, *v*/*v*). The mixture was then poured into a filter, allowing gravity to separate the oil and water phases [33]. The results demonstrated oil sorption capacities ranging from 43 g/g to 106 g/g.

**Figure 2:**
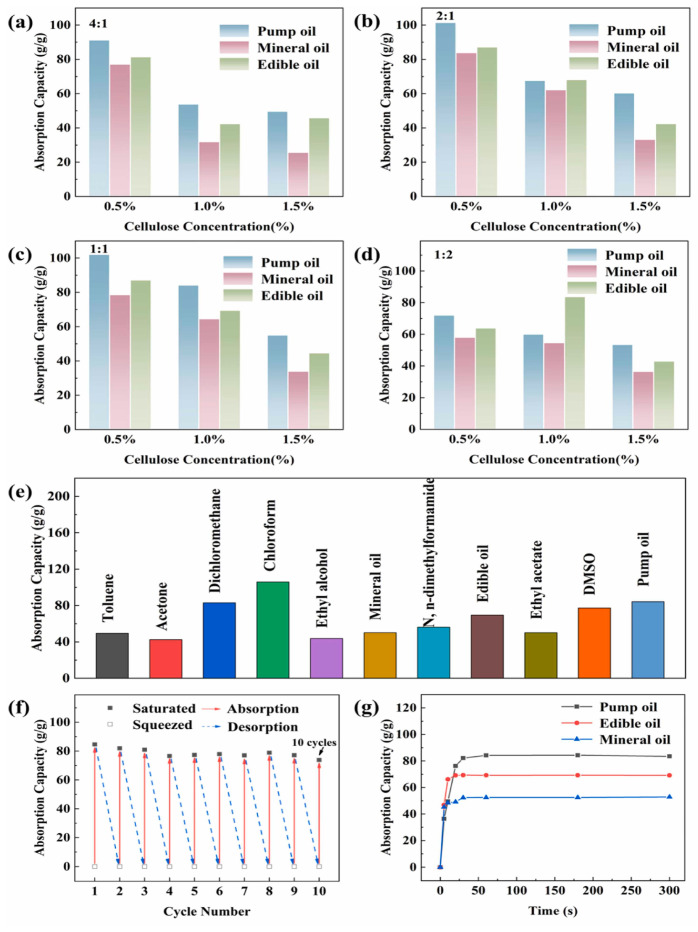
Sorption capacity of prepared aerogels for pump oil, mineral oil, and edible oil (**a**–**d**); sorption capacity for different oils and organic solvents (**e**); reusability test for cyclic sorption of pump oil (**f**); sorption capacity vs. time (**g**). Source [33] with permission from the Elsevier and Copyright Clearance Center.

Figure 2a–d illustrates sorption capacities for pump, mineral, and edible oils, while Figure 2e presents sorption data for various oils and organic solvents. The cyclic sorption performance for pump oil and sorption rate over time are shown in Figure 2f,g. Notably, the results indicated that the aerogels could achieve oil/water separation efficiencies exceeding 98% in continuous operations, highlighting their potential for effective environmental applications [33].

Ma and collaborators [28] further explored aerogel synthesis using a blend of kapok fibers (KF) with regenerated cellulose derived from hardwood pulp, producing a material labeled KRxCA. Among the synthesized samples, KR10CA, containing 10% regenerated cellulose, exhibited remarkable structural integrity, maintaining its original shape and volume to a significant extent even after undergoing carbonization at 400 °C [28]. Subsequently, KR10CA was tested for its sorption capabilities with various oils (pump oil, paraffin oil, olive oil, and hydraulic oil) and organic solvents (n-hexane, acetone, carbon tetrachloride, dichloromethane, and dimethyl sulfoxide) [28]. The aerogel exhibited remarkable sorption capacities, ranging from 137.5 g/g to 371.7 g/g for the tested oils and solvents [28]. Notably, KR10CA retained 89.3% of its sorption capacity even after 10 cycles of use, indicating its durability and efficiency. In addition to its sorption capabilities, KR10CA demonstrated outstanding performance in separating oil from water mixtures, achieving a separation efficiency of 99.95% or greater [28]. The aerogel also showcased exceptional hydrophobic properties, reflected in a high contact angle of 144.7°, further underscoring its potential applications in environmental remediation and industrial processes [28].

#### 2.3.3. Hybrid Aerogels Based on Cellulose and Nano-Polysaccharides

Hybrid aerogels derived from nano-polysaccharides, chitin nanocrystals (ChiNC), and TEMPO-oxidized cellulose nanofibers (TCNF), as well as their derivative cationic guar gum (CGG), have demonstrated remarkable potential for oil and organic solvent sorption. Yagoub and co-workers [57] successfully developed a superhydrophobic/superoleophilic aerogel (ChiNC/TCNF/CGG) using a freeze-drying method with glutaraldehyde (GA) as a cross-linker. This aerogel exhibited impressive sorption capacities for various oils and solvents, including corn oil (6.8 g/g), n-hexane (9.4 g/g), toluene (12.6 g/g), and trichloromethane (21.9 g/g) [57]. Additionally, the aerogel demonstrated excellent reusability for up to 10 cycles while maintaining its sorption efficiency. The effectiveness of the modified aerogel was validated by immersing it in an oil/organic solvent/water mixture, where it efficiently separated the two phases [5].

In another study, Ye and co-workers [58] optimized the development of a bagasse-derived cellulose nanofiber aerogel using factorial design, incorporating sodium alginate and calcium carbonate. The cellulose nanofibers were extracted from bagasse, yielding an aerogel with a water contact angle (WCA) of 135 °C, extremely high porosity (99.47%), and an oil/solvent sorption capacity ranging from 59 g/g to 126 g/g [58]. This aerogel successfully absorbed a wide range of organic pollutants, including tetrachloromethane, soybean oil, chloroform, benzene, diesel, dichloromethane, simethicone, pump oil, octane, and n-hexane [58]. A reusability study showed that the diesel sorption capacity remained at 112 g/g after the first cycle and gradually decreased to 93 g/g after 10 cycles, demonstrating significant long-term efficiency [58]. The aerogel’s practical performance was evaluated in diesel/water and tetrachloromethane/water mixtures, where it successfully removed diesel in just 20 s and tetrachloromethane in 15 s [58].

Chen and collaborators [59] reported the preparation of an eco-friendly aerogel derived from *Willow moss*, which exhibited a WCA of 148 °C and outstanding sorption capacities for n-hexane, petroleum, diesel, toluene, soybean oil, dichloromethane, and CCl_4_ (22.45–67.23 g/g) [59]. Furthermore, this aerogel demonstrated excellent reusability over 10 cycles while maintaining its efficiency [54]. Notably, it achieved an impressive 99.9% separation efficiency for emulsions, highlighting its potential for large-scale environmental applications [59].

Another notable aerogel, designed for marine environments due to its salt tolerance and excellent oil retention, was developed by lyophilizing sodium alginate with cellulose nanofibers cross-linked with Ca^2+^ ions [60]. The synthesis involved ultrasonication of cellulose to ensure uniform dispersion, followed by dissolution of sodium alginate in deionized water to form a transparent solution. These two suspensions were combined and magnetically stirred until a homogeneous mixture was obtained. The resulting solution was frozen at −15 °C and lyophilized under reduced pressure (400 µbar), forming a primary aerogel. To enhance its stability and practical utility, the aerogel was further cross-linked with calcium chloride, washed thoroughly to remove excess calcium ions, and lyophilized again to consolidate its structure. This sodium alginate/nanofibrillated cellulose aerogel exhibited strong oleophilic properties, high robustness, and long-term stability in marine environments. Its sorption efficiency exceeded 99%, and it retained its effectiveness over 40 sorption–desorption cycles. This high-performance aerogel represents a significant advancement in sustainable materials for oil spill remediation and wastewater treatment.

### 2.4. Hybrid Aerogels

Hybrid aerogels represent an advanced class of materials designed for efficient oil sorption and environmental remediation. Their highly porous structures, combined with exceptional hydrophobicity and oleophilic properties, make them ideal candidates for oil spill cleanup, wastewater treatment, and separation of organic pollutants from water.

#### 2.4.1. Kapok Fiber–Silica Nanoparticle Composite for Oil Sorption

A highly hydrophobic composite material was successfully synthesized using the sol-gel method, embedding silica nanoparticles into kapok fiber to enhance its oil sorption capabilities [61]. The kapok fiber was first treated with a 0.5 wt% sodium chlorite solution, and its pH was adjusted to 4.5 using acetic acid. After mechanical stirring, the fiber was thoroughly washed with distilled water until a neutral pH was achieved and subsequently dried to a constant weight [61]. To introduce silica into the fiber matrix, tetraethyl orthosilicate (TEOS, 4 wt%) and sodium dodecyl benzene sulfonate (1.2 mmol/L) were added to distilled water and stirred to form a uniform solution [61]. The pre-treated fibers were then immersed in this mixture, followed by the addition of ammonium hydroxide to facilitate silica deposition. After 4 h of reaction, the fibers were thoroughly washed with methanol, dried at 60 °C, and further hydrolyzed in dodecyltrimethoxysilane to enhance their hydrophobicity [61]. Finally, the modified fibers were filtered, dried, and solidified at 120 °C to obtain the final composite material [61]. The resulting hydrolyzed dodecyltrimethoxysilane-treated kapok fiber exhibited superior hydrophobicity and was particularly effective for oil and diesel sorption, demonstrating its potential for environmental applications [61].

#### 2.4.2. Silylated Bacterial Cellulose Aerogels (SBCAs) for Oil Sorption

**Figure 3:**
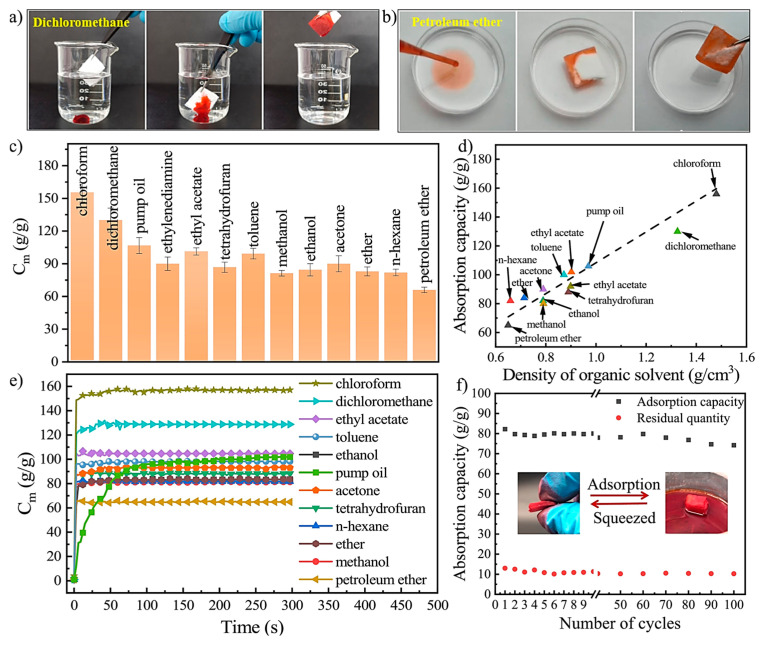
Oil sorption and separation performance of SBCA1. (**a**,**b**) Removal of dichloromethane (colored with Sudan III) from the bottom of water and petroleum ether (colored with Sudan III) on the water surface with a piece of SBCA1. (**c**) Mass-based sorption capacities of SBCA1 for various oils and organic solvents. (**d**) Sorption capacities for different organic solvents as a function of the liquid density. (**e**) Sorption kinetics curves of SBCA1. (**f**) Cyclic sorption capacities of the SBCA1 for n-hexane. Source [62] with permission from Elsevier and Copyright Clearance Center.

In another study, Ke and colleagues [62] successfully developed silylated bacterial cellulose aerogels (SBCAs), producing three variants: SBCA1, SBCA2, and SBCA3. These aerogels exhibited outstanding porosity (~99%) and remarkable hydrophobicity, with water contact angles (WCAs) exceeding 120° [62]. The enhanced hydrophobicity was attributed to the silylation process, which improved the aerogels’ ability to repel water while maintaining their oleophilic nature [62]. The structural and performance characteristics of these hybrid aerogels are summarized in Figure 3, highlighting their highly porous network and superior sorption capacity for organic pollutants. These findings demonstrate the potential of silylated bacterial cellulose aerogels as efficient oil absorbents, offering promising applications in oil spill remediation and wastewater treatment [62].

### 2.5. Graphene Oxide-Based Aerogels

Most three-dimensional macrostructures designed for water and air treatment have a spongy structure and form hydrogels, aerogels, and xerogels [63]. Among them, graphene oxide (GO) has emerged as an exceptional precursor for the synthesis of adsorbents, primarily due to its high Brunauer–Emmett–Teller (BET) surface area and tunable surface chemical composition. Graphene-based aerogels are particularly promising as sorption materials owing to their intrinsic properties, such as low density, elevated specific surface area, and significant porosity [64]. Guo and collaborators [65] emphasized that graphene aerogels harness the synergistic advantages of both graphene and aerogel, making them highly effective for sorption applications.

#### 2.5.1. Oil/Water Separation Performance of Graphene-Based Aerogels

**Figure 4:**
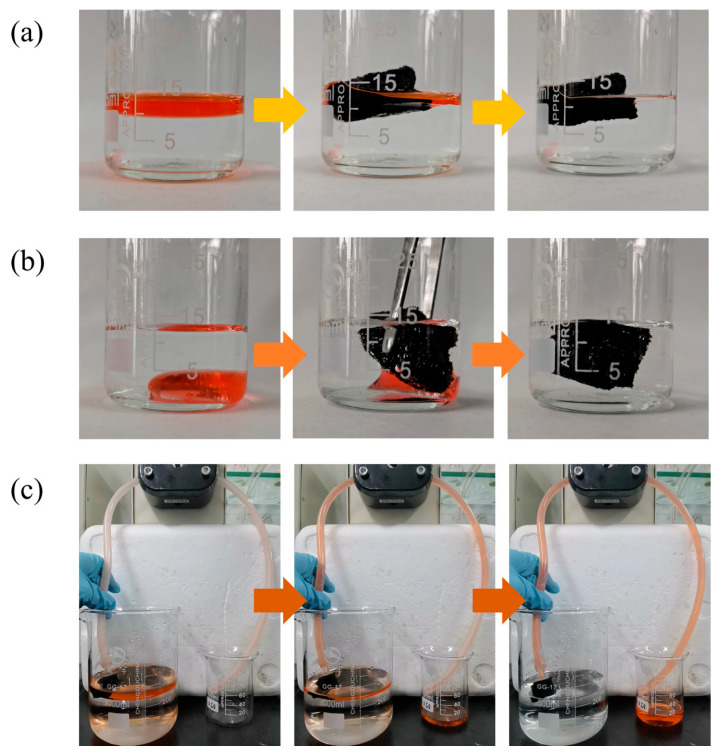
Oil−water separation performance of GKM-2. (**a**) Absorption process of cyclohexane; (**b**) absorption process of dichloromethane; and (**c**) GKM-2 continuously separated the cyclohexane from water. Source [30].

Shen et al. [30] investigated the oil/water separation efficiency of a graphene-based aerogel, GKM-2, demonstrating its rapid sorption of organic solvents such as cyclohexane (Figure 4a) and dichloromethane (Figure 4b). Notably, GKM-2 continuously separated cyclohexane from water (Figure 4c), underscoring its potential for efficient oil recovery [30].

**Figure 5:**
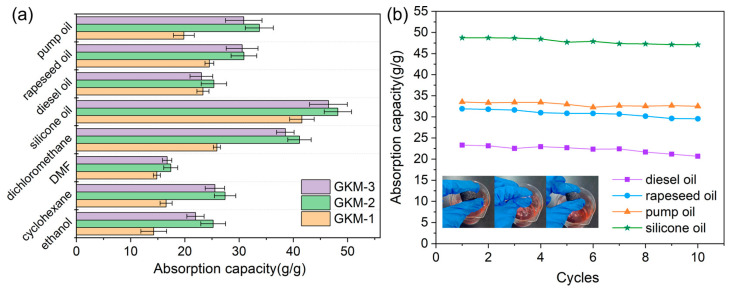
Oil absorption and regeneration performance of GO@KGM aerogels. (**a**) Oil absorption capacity histogram; (**b**) 10 absorption cycles for pump oil, rapeseed oil, diesel oil, and silicone oil by GKM-2. Source [30].

This eco-friendly biomass aerogel was developed using konjac glucomannan (KGM) as the primary raw material, incorporating graphene oxide (GO) via a freeze-drying technique. The successful cross-linking of KGM and GO endowed the aerogel with exceptional mechanical strength and directional oil sorption, making it highly effective for oil spill remediation [30]. To further enhance its hydrophobicity, the aerogel was surface-modified with methyltrimethoxysilane (MTMS), significantly improving its water resistance and enabling efficient oil/water separation. The composite polysaccharide-based aerogel exhibited remarkable oil sorption, retaining up to 48 times its own weight [30]. Moreover, the aerogel demonstrated outstanding mechanical resilience and reusability, maintaining a 96% recovery rate even after 10 cycles (Figure 5).

Its ability to continuously absorb oil from water highlights its practical applicability in diverse environmental scenarios [30]. Overall, these findings position KGM-based aerogels as a promising, sustainable solution for large-scale oil/water separation, offering high efficiency, durability, and significant environmental benefits [30].

#### 2.5.2. Corn Stalk-Derived Graphene Aerogels (CSGA) for Oil Sorption

**Figure 6:**
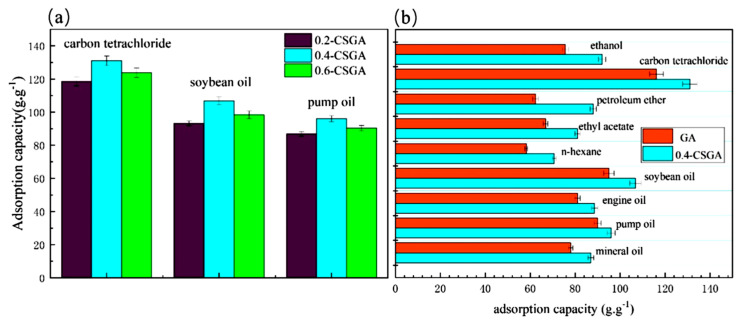
Sorption capacities of 0.2−CSGA, 0.4−CSGA, and 0.6−CSGA (**a**); Comparison of sorption capacities between GA and 0.4−CSGA for various organic reagents (**b**). Source [66] with permission from the Elsevier and Copyright Clearance Center.

Zhang and co-workers [66] synthesized CSGA aerogels from natural corn stalk powder and graphene oxide (GO). Three aerogels were developed with varying corn stalk (CS)-to-graphene aerogel (GA) mass ratios: 0.2/1, 0.4/1, and 0.6/1 [66]. The oil sorption test was conducted for carbon tetrachloride, soybean oil, and pump oil (Figure 6a), while a comparison of GA and 0.4-CSGA sorption capacities for various organic solvents (ethanol, carbon tetrachloride, petroleum ether, ethyl acetate, n-hexane, soybean oil, engine oil, pump oil, and mineral oil) is shown in Figure 6b [66].

The reusability of 0.4-CSGA was evaluated over eight sorption–desorption cycles for soybean oil, pump oil, engine oil, and mineral oil. Notably, 0.4-CSGA demonstrated ultrafast sorption performance, achieving complete sorption of carbon tetrachloride and pump oil within 3 s [66].

#### 2.5.3. Straw-Based Graphene Aerogels (SGA) for Oil/Water Separation

Crop straw materials, particularly wheat straw (WS), were utilized to fabricate straw-based graphene aerogels (SGA) for oil/water separation [67]. After acidic and alkaline pre-treatments, the wheat straw was cross-linked with GO to form different WS-based aerogels [67].

(i) WSGA refers to the wheat straw-based graphene aerogel;
(ii) AC-WSGA denotes the aerogel derived from acid-treated wheat straw;
(iii) AL-WSGA represents the aerogel obtained from alkaline-treated wheat straw.

The sorption capacities of these aerogels for various substances, including phenixin, toluene, n-hexane, engine oil, and peanut oil, were meticulously assessed. The findings revealed sorption capacities ranging from 37 g/g to 98.7 g/g for WSGA, 62.3 g/g to 126 g/g for AC-WSGA, and 66.3 g/g to 125.2 g/g for AL-WSGA [67]. To comprehensively evaluate the performance of the aerogels, the authors included several critical analyses, such as (i) the sorption capacity results, which were plotted against time (in seconds) for all three aerogels, (ii) a recyclability test, which was conducted for each aerogel through methods including heat treatment and solvent replacement, and (iii) the recyclability of WSGA and AL-WSGA, which was examined using an extrusion technique [67]. These investigations provide valuable insights into the efficacy and sustainability of straw-based graphene aerogels in practical applications for oil/water separation [67].

#### 2.5.4. Innovations in Graphene Aerogels

##### Graphene/Carbon Nanotube (CNT) Aerogels with Superhydrophobicity and Oil Sorption

In another innovative approach, Zhao et al. [68] prepared porous graphene/carbon nanotube aerogels through hydrothermal reduction, freeze-drying, and high-temperature treatment, using ascorbic acid as a reducing agent. These aerogels exhibited impressive compression resilience and superhydrophobic properties, particularly when the carbon nanotube (CNT) content exceeded 50%, with demonstrated oil affinity reaching up to 100 g/g [67]. The resulting materials were characterized as ultralight, with a density of only 3 mg/cm^3^ [68].

##### Graphene Aerogels via Solvothermal Reduction for Oil/Organic Solvent Separation

Pruna and collaborators [69] conducted an insightful investigation into the influence of surface chemistry of graphene oxide (GO) on its gelation properties through a combined chemical/thermal reduction approach. This study also evaluated the subsequent effects on the properties and sorption capacity of the resulting aerogels [69]. Notably, ascorbic acid was employed as a reducing agent for the graphene oxide during the process [68]. To synthesize the aerogel, a previously ultrasonicated aqueous suspension of GO was treated with ascorbic acid at elevated temperatures reaching up to 165 °C. This treatment was followed by dehydration at −20 °C to facilitate gel formation [69]. The resultant hydrogels were transformed into three-dimensional porous aerogels through a meticulous freeze-drying process [69]. This involved cooling the hydrogels at −80 °C under high vacuum conditions, utilizing three-directional cooling, followed by sublimation at 20 °C for 48 h at a low pressure of 0.015 mbar [69]. The sorption properties of these aerogels were rigorously assessed using engine oil and dichloromethane as test fluids. The findings revealed that the aerogels exhibited superior sorption capabilities, particularly when characterized by a lower carbon-to-oxygen (C/O) ratio of GO [69]. Remarkably, the optimal aerogels achieved an impressive oil uptake capacity of 86 g/g, highlighting their potential for applications in oil spill remediation and environmental clean-up efforts [69].

##### Biomimetic Graphene/Polyvinyl Alcohol (PVA)/Cellulose Nanofiber Aerogels

Feng et al. [70] reported significant advancements in the development of highly compressible aerogels composed of graphene oxide (GO), TEMPO-oxidized cellulose nanofibers, and polyvinyl alcohol (PVA) through an innovative combination of bidirectional freezing and chemical vapor deposition (CVD). These advanced aerogels demonstrated impressive sorption capacities ranging from 75 g/g to 151 g/g, contingent upon the specific organic solvent utilized [70]. Notably, the aerogels exhibited remarkable recovery efficiency, achieving oil retention rates of up to 91.5% even after undergoing multiple cycles of sorption and compression [70]. This outstanding performance highlights their promising potential for practical applications in oil removal and environmental remediation [70].

Zhang and collaborators [71] developed innovative porous graphene oxide/polyimide (GO/PI) aerogels through a meticulous process starting with mixed suspensions of water-soluble polyimide and graphene oxide sheets. These suspensions underwent a freeze-drying process, followed by thermal imidization, resulting in aerogels with remarkable properties [71]. Notably, these aerogels can support an astonishing weight of up to 31,250 times their own mass. Their sorption capacity for oil and organic solvents—such as Arawana cooking oil, ethanol, and glycerol—ranges impressively from 14.6 to 85 times their weight [71]. The exceptional flexibility and high compressive strength of these aerogels can be attributed to their unique honeycomb-like structures and the dense arrangement of non-covalent interactions, which include sacrificial bonds and strong interfacial interactions [71].

### 2.6. Other Aerogel Materials

In addition to graphene-based systems, various other aerogel materials have been explored for oil/organic solvent sorption.

#### 2.6.1. Cross-Linked Gelatin Aerogels for Organic Pollutant Removal

Wang and collaborators [72] conducted a comprehensive investigation into the efficacy of cross-linked gelatin aerogels (MTCS-cGel) for the sorption of various organic solvents and oils, including toluene, chloroform, paraffin oil, waste pump oil, pump oil, silicone oil, kerosene, gasoline, and crude oil [71]. Characterized by a high porosity of 95.01%, the MTCS-cGel demonstrated remarkable sorption capacities, ranging from 70 g/g to 123 g/g [72]. Furthermore, a reusability study involving 50 cycles was carried out to assess the performance of the aerogel, specifically for gasoline, employing a compression method. The findings strongly endorse the potential of the developed sorption aerogel as an effective solution for the removal of oil contaminants, highlighting its promising applications in environmental cleanup efforts [72].

#### 2.6.2. Superhydrophobic Polyacrylonitrile (PAN)/Polybenzoxazine (PBOZ) Aerogels

**Figure 7:**
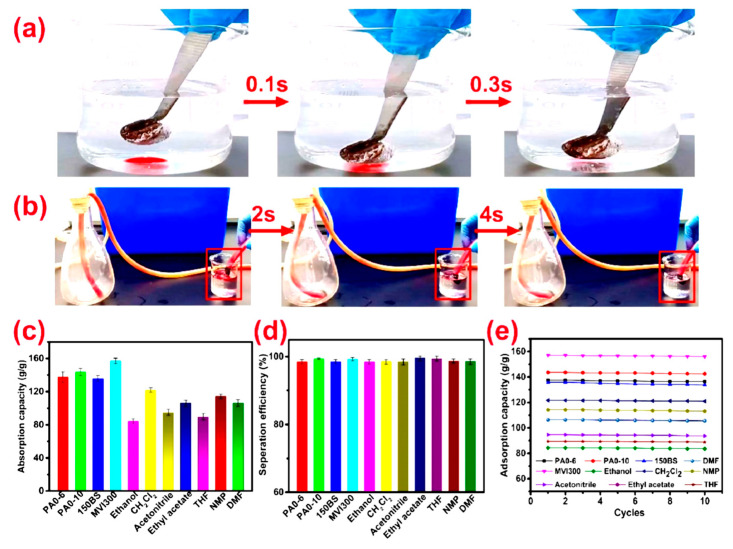
Photographs of chloroform (**a**) and n-hexane (**b**) sorption from water by PAN/PBOZ aerogel, respectively; Sorption capacity of PAN/PBOZ for different organic solvents or oils (**c**); Separation efficiency after 10 cycles for PAN/PBOZ (**d**); Sorption capacity of PAN/PBOZ at different cycles (**e**). Source [73] with permission from the Elsevier and Copyright Clearance Center.

Xu and colleagues [73] successfully developed a superhydrophobic polyacrylonitrile/polybenzoxazine aerogel (PAN/PBOZ) that exhibits impressive water contact angle (WCA) and porosity values of 156° and 98.5%, respectively. The experimental results indicate that the PAN/PBOZ fiber aerogel demonstrates rapid sorption of carbon tetrachloride (CCl_4_), as illustrated in Figure 7a [73]. Similarly, n-hexane is sorbed at a remarkably fast rate, as shown in Figure 7b. The sorption capacities for these targeted pollutants vary significantly, ranging from 84.3 g/g to 157.7 g/g, as depicted in Figure 7c [73]. Furthermore, the separation efficiency and sorption capacity of the PAN/PBOZ aerogel after 10 cycles of use are presented in Figure 7d,e, highlighting its potential for sustainable and efficient pollutant removal applications [73].

Table 2 provides a concise overview of additional studies focused on the investigation of aerogel-type materials. These studies utilized various starting materials in their synthesis processes, specifically aimed at exploring oil and organic solvent sorption capabilities [73].

#### 2.6.3. Aerogels Derived from Recycled Pollutants

One of the most significant environmental threats today is the widespread disposal of plastic bottles, primarily made from polyethylene terephthalate (PET), which can take hundreds of years to biodegrade. A promising approach to combat this issue is the development of aerogels derived from recycled PET fibers, which not only provides a sustainable waste management strategy but also offers effective applications in wastewater treatment [56,80]. The fabrication process of these aerogels involves multiple technological stages. Initially, PET fibers are immersed in a sodium hydroxide solution and heated to 80 °C for one hour, facilitating the formation of carboxyl and hydroxyl groups on their surface [56]. After thorough washing with deionized water, the fibers are submerged in a mixture of polyvinyl alcohol, glutaraldehyde, and deionized water. The reaction can be accelerated by introducing hydrochloric acid [56]. Subsequently, the material undergoes sonication, further heating at 80 °C, freezing, and lyophilization [56]. To enhance hydrophobicity, the dried aerogel is coated with methyltrimethoxysilane and subjected to an additional drying step at 70 °C [56]. The resulting aerogels exhibit ultra-low density, an average pore size of approximately 50 nm, and porosity exceeding 98% [56]. These properties are significantly influenced by the PET fiber content. At a PET concentration of 0.5% by weight, the aerogel achieves an optimal density of 0.011 g/cm^3^ and a porosity of 99.2%, demonstrating a remarkable sorption capacity of 70.2 g/g [56]. This innovative approach not only provides a sustainable solution for PET waste management but also presents an efficient material for environmental applications, particularly in wastewater treatment and pollutant removal [56].

In a recent study conducted by Tu et al. [80], a novel aerogel was synthesized from waste plastic bags (PBA) and subsequently modified with graphene oxide (GO) to enhance its mechanical strength, resulting in the composite known as GO/PBA [80]. Further modifications involved coating this composite with poly(dimethylsiloxane) (PDMS), leading to the creation of PDMS/GO/PBA, which was specifically designed to improve selectivity in oil/water separation applications [80]. The water contact angles (WCAs) measured at 103° for PDMS/PBA and 105° for PDMS/GO/PBA indicate a high degree of hydrophobicity for both materials [80]. The aerogels demonstrated impressive sorption capacities for crude oil, with PDMS/PBA exhibiting a capacity of 16 g/g, while PDMS/GO/PBA significantly outperformed it with a capacity of 27 g/g. Notably, both aerogels achieved equilibrium in their sorption processes within just 5 s, showcasing their rapid efficiency [80]. Furthermore, the PDMS/GO/PBA aerogel was rigorously tested in an oil/water system, where it successfully completed oil sorption in approximately 10 s. To evaluate the practicality of this material, a reusability test was conducted over 10 cycles using the sorption–squeezing method in diesel oil, affirming the aerogel’s potential for repeated use in real-world applications [80].

The development of aerogels from recycled PET fibers and other plastic waste represents a significant step forward in addressing environmental pollution while simultaneously creating valuable materials for ecological remediation. With their ultra-low density, high porosity, and impressive sorption capacities, these aerogels are poised to play a crucial role in enhancing wastewater treatment processes and mitigating the impact of plastic waste.

### 2.7. Mechanisms and Optimization Strategies for Oil, Fat, and Grease (FOG) Removal from Wastewater

Removing oils, fats, and greases (FOGs) from wastewater is crucial to modern water treatment, as these hydrophobic pollutants pose significant environmental and operational challenges. Due to their complex physicochemical properties, the sorption process remains one of the most effective strategies for their removal, driven by a series of well-defined mechanisms. These mechanisms can be broadly categorized into diffusion, retention, and fixation phases, each governed by distinct chemical and physical interactions. A comprehensive understanding of these processes is essential for optimizing sorption efficiency, enhancing the performance of adsorbent materials, and advancing the development of sustainable wastewater treatment technologies.

#### 2.7.1. Mechanism of FOG (Fats, Oils, and Grease) Removal Using Aerogels

Aerogels are effective materials for FOG removal due to a combination of physical and chemical interactions, along with advantageous structural properties [5,24,38,46,51,55,56,72,78]. The key mechanisms are described below.

##### Physical Sorption

This process relies on non-covalent interactions between the aerogel and the FOG molecules. Specifically, van der Waals forces and hydrophobic interactions contribute to the adhesion of oil molecules to the pore walls. The FOG molecules diffuse into the porous network and are retained through these interactions with the walls of the pores [5,24,38,46,51,55,56,72,78].

##### Chemical Sorption

The sorption capacity of aerogels can be significantly enhanced through chemical modification. By incorporating specific functional groups onto the aerogel’s surface, the material can establish stronger, more specific interactions with FOG molecules. These interactions may include hydrogen bonding, dipole–dipole interactions, or other chemical bonds between the modified aerogel and polar components found in some oils or grease. This modification strategy can significantly increase the retention capacity of the aerogel [5,24,38,46,51,55,56,72,78].

##### Diffusion and Transport

The high porosity of aerogels is crucial for accelerating the diffusion of FOG molecules into their internal structure. This enhanced diffusion contributes to the kinetics of the sorption process, allowing for a faster uptake rate of contaminants. Nanoscale pore sizes can minimize diffusion limitations, allowing for rapid uptake of contaminants [5,24,38,46,51,55,56,72,78].

##### Coalescence and Flocculation

Aerogels can act as a surface for the coalescence of dispersed oil droplets. The porous structure traps smaller oil or grease droplets, bringing them into close proximity. This allows the droplets to merge and form larger aggregates, facilitating their subsequent removal through processes such as skimming or filtration [5,24,38,46,51,55,56,72,78].

##### Macroscopic Oil Collection (Floating and Concentration)

Due to their lightweight and hydrophobic nature, aerogels can float on the surface of water, allowing them to capture and concentrate oils floating on the water surface. This characteristic is particularly useful for oil spill cleanup and treatment of oily wastewater. The absorbed oil can then be easily recovered from the aerogel, making it a practical and efficient solution [5,24,38,46,51,55,56,72,78].

##### Effect of Density and Thickness

The density and thickness of the aerogel layers also affect sorption performance. Thinner layers may provide faster kinetics, leading to quicker initial uptake. Conversely, thicker aerogel layers can provide a higher overall sorption capacity but may introduce diffusion limitations, which can slow down the overall process and diminish the rate of sorption. The optimal layer characteristics (density, thickness) depend on the specific application and the desired balance between sorption rate and capacity [5,24,38,46,51,55,56,72,78].

### 2.8. Insight into Mechanisms of FOG Sorption—A Three-Stage Process

#### 2.8.1. Diffusion and Surface Interaction

The initial phase of FOG removal involves the diffusion of oil, fat, and grease molecules toward the surface of an adsorbent. These substances are retained by a blend of intramolecular forces, van der Waals interactions, and the chemical compatibility of the adsorbent and the adsorbate. The porous architecture of the adsorbent, combined with capillary forces, enhances this phase of these organic contaminants. This process is primarily influenced by molecular motion and environmental conditions, such as temperature and viscosity [5,24,38,46,51,55,56,72,78].

Retention at the surface occurs via a combination of physical and chemical interactions.

##### Physical Interactions

Van der Waals forces, capillary action, and hydrophobic interactions facilitate the adhesion of FOG molecules onto the adsorbent. Adsorbents with high surface roughness and hydrophobicity enhance this process [23]. The predominant physical bonding mechanisms include the following:

(a) Van der Waals forces contribute to the adhesion of non-polar FOG molecules (such as hydrocarbons) to the surface of the aerogel. The high surface area provided by the porous structure of aerogels maximizes these interactions, facilitating the sorption process [22,23,24].
(b) Hydrophobic interactions arise when non-polar substances aggregate to minimize their exposure to water. This effect is prominent in the presence of hydrophobic materials. The hydrophobic aerogels modified with long-chain alkyl groups have a strong affinity for non-polar FOGs [22,23,24]. The hydrophobic nature of the aerogel surface reduces the surrounding water layer’s stability, encouraging the sorption of oils and fats [22,23,24].
(c) Capillary action occurs whereby liquids rise or fall in narrow openings against the force of gravity due to surface tension. As FOG droplets are introduced to the porous structure, capillary action can pull the liquids into the pores of the aerogel, promoting sorption and subsequent retention [22,23,24].

##### Chemical Interactions

Beyond the physical confines of surface area and pore size, the chemical nature of the adsorbent and the organic functional group (FOG) molecules plays a critical role in dictating sorption performance [5,24,38,46,51,55,56,72,78].

This interaction is a crucial factor in determining the strength and selectivity of the sorption process, essentially acting as a molecular “glue” that draws FOGs to the adsorbent surface. The presence of specific functional groups on the adsorbent surface can create strong attractive forces with FOG molecules, dramatically enhancing their affinity and retention [5,24,38,46,51,55,56,72,78]. This includes the following:

(i) Hydrogen bonding

The functional groups present on the adsorbent’s surface, such as hydroxyl (-OH) or amino (-NH_2_) groups, are highly effective in forming hydrogen bonds with FOG molecules containing oxygen (e.g., -OH, C=O) or nitrogen (e.g., -NH_2_) atoms. These hydrogen bonds can significantly increase the sorption strength, arising from the attraction between a partially positive hydrogen atom and a partially negative oxygen or nitrogen atom. This process can be likened to a network of weak but abundant molecular interactions, collectively ensuring the effective adhesion of FOG to the surface [81,82,83,84,85,86,87].

(ii) Dipole–dipole interactions can occur between FOGs with polar characters and aerogels with polar surface groups, strengthening the adsorptive binding [55,81,82,83,84,85,86,87,88,89,90].
(iii) Ionic interactions are the attraction of opposites. If the adsorbent surface possesses charged functional groups, such as carboxyl (-COOH) groups that can deprotonate to form carboxylate ions (-COO^−^) or amine groups (-NH_3_^+^) that can gain a proton, it can establish electrostatic interactions, also known as ionic interactions, with FOG molecules bearing opposite charges. This is particularly relevant when dealing with FOGs that are ions or can be ionized under specific pH conditions. The stronger the charge and the closer the proximity, the stronger the attraction [55,81,82,83,84,85,86,87,88,89,90].
(iv) Ion–dipole interactions occur when, in the presence of ionic substances (such as salts), aerogels may form ion–dipole interactions with charged species in the water, potentially enhancing the overall interaction with oily materials [55,81,82,83,84,85,86,87,88,89,90].
(v) π–π interactions occur in cases where both the adsorbent and the FOG molecules contain aromatic rings (e.g., benzene rings); π–π interactions can play a significant role. These interactions arise from the overlapping of the electron clouds in the pi systems of the aromatic rings, leading to a weaker, but still significant, attraction [55,81,82,83,84,85,86,87,88,89,90].

#### 2.8.2. Retention in Porous Structure

Once the FOG molecules reach the adsorbent surface, they penetrate the porous structure and begin to fill the available voids within the material. The size and distribution of these pores are crucial for maximizing oil retention, as they determine how effectively the adsorbent can trap the oil. Stronger physical bonds in this stage, such as hydrophobic interactions between hydrophobic FOG molecules and hydrophobic surface areas of the adsorbent, can promote considerable retention capacity. Additionally, chemical interactions can occur if the adsorbent contains reactive sites that can bond with the fat and grease molecules, enhancing overall binding strength [55,81,82,83,84,85,86,87,88,89,90].

(i) *Pore size effect*: Micropores (<2 nm) and mesopores (2–50 nm) contribute to enhanced sorption by trapping smaller oil droplets, while macropores (>50 nm) facilitate bulk transport [85].
(ii) *Hydrophobicity*: Hydrophobic interactions between non-polar FOG molecules and hydrophobic regions of the adsorbent prevent desorption, ensuring prolonged retention [86].

#### 2.8.3. Fixation and Long-Term Retention

Finally, the oil becomes fixed within the pores of the adsorbent. This fixation is influenced by various factors, including the interactions between the oil and the adsorbent material, leading to a stable and effective sorption process [55,81,82,83,84,85,86,87,88,89,90]. The stability of this fixation depends on the strength of bonding interactions.

(i) *Physical retention*: Stronger van der Waals forces and capillary action can immobilize FOGs in high-surface-area adsorbents [81,84,85].
(ii) *Chemical fixation*: Functionalized adsorbents with carboxyl or amino groups can form covalent or electrostatic bonds, significantly enhancing sorption strength and preventing desorption under variable environmental conditions [81,83,87,88].

#### 2.8.4. Examples Illustrating Chemical Synergy

Activated carbon and biochar: The excellent sorption capabilities of activated carbon and biochar are, in part, attributed to their oxygen-containing functional groups (e.g., hydroxyl, carbonyl, carboxyl). These groups enable the formation of strong hydrogen bonds with a wide array of FOG molecules, making them effective adsorbents for various pollutants [84,85,86].

#### 2.8.5. Combined Mechanisms

In many cases, the interaction between FOGs and aerogels involves a combination of both physical and chemical bonding mechanisms [5,24,38,44,51,55,81,82,83,85,89]. As FOGs are adsorbed onto the aerogel surface, multiple types of interactions can occur simultaneously.

(v) *Sequential mechanisms*: Initial sorption may occur predominantly via physical methods (van der Waals forces and hydrophobic interactions), followed by stronger chemical bonding (like hydrogen bonding or dipole–dipole) as the FOGs migrate further into the aerogel’s porous structure [5,24,38,44,51,55,81,82,83,85,89].
(vi) *Layered mechanism*: The first molecules of oil may be adsorbed onto the aerogel surface via hydrophobic and van der Waals forces, which subsequently promotes the binding of other molecules through additional hydrogen and dipole interactions [5,24,38,44,51,55,81,82,83,85,89].

A recent review paper on aerogels for water treatment [19] emphasized that the chemisorption with ion exchange as a mediating process is the basis of the mechanism of sorption. Generally, the sorption data follow the Langmuir isotherm, while the Pseudo second-order kinetic model describes the reaction kinetics of sorption.

### 2.9. Aerogels—Tailoring the Surface for Targeted Sorption

With their highly porous structures, aerogels offer vast surface areas for interaction. Their inherent flexibility in surface chemistry further enhances their sorption performance [5,24,38,44,51,55,81,82,83,85,89]. For example, the incorporation of functional groups like hydroxyl (-OH) or carboxyl (-COOH) groups onto the aerogel surface allows for the formation of hydrogen bonds or electrostatic interactions with FOG molecules, substantially improving their sorption strength [5,24,38,44,51,55,81,82,83,85,89]. This targeted functionalization allows for the creation of tailored adsorbents with selectivity for specific FOGs [5,24,38,44,51,55,81,82,83,85,89]. Understanding and strategically incorporating specific functional groups on adsorbent materials is a powerful approach to enhancing sorption performance. These chemical interactions offer a level of control and specificity beyond simple physical sorption, enabling the development of highly effective and selective adsorbents for a variety of environmental and industrial applications [5,24,38,44,51,55,81,82,83,85,89]. By carefully selecting and modifying the surface chemistry of the adsorbent, we can finely tune the interactions to maximize the capture and retention of FOGs [5,24,38,44,51,55,81,82,83,85,89].

### 2.10. Retention in Porous Structures

Once adsorbed, FOG molecules penetrate into the porous structure of the material. The retention capacity is significantly influenced by pore size, distribution within the adsorbent, and surface chemistry, as these characteristics dictate how well the contaminants can be retained [5,24,38,44,51,55,81,82,83,85,89].

### 2.11. Factors Influencing FOG Removal Efficiency

Several environmental and operational factors affect the efficiency of sorption-based wastewater treatment [5,24,38,44,51,55,81,82,83,85,89].

#### 2.11.1. pH Effects

The pH of wastewater significantly alters the sorption capacity of different materials by influencing surface charge and chemical stability. Many adsorbent materials possess functional groups that can become charged depending on the pH of the solution. For example, acidic conditions enhance the sorption of negatively charged fatty acids due to the protonation of adsorbent functional groups [5,24,38,44,51,55,81,82,83,85,89]. Conversely, in alkaline pH (>7), deprotonation may occur, weakening electrostatic interactions between the adsorbent and the negatively charged molecules and reducing sorption efficiency [23]. pH can also affect the chemical stability of some adsorbents. For instance, metal oxides can dissolve at extreme pH levels, compromising their performance [17,23,24,90]. Studies have indicated that optimal pH values can vary among different adsorbents. For instance, activated carbon might perform best at a slightly alkaline pH, while biochar might exhibit higher sorption efficiencies in slightly acidic conditions [17,23,24,90].

#### 2.11.2. Contaminant Concentration and Sorption Isotherms

The initial concentration of OFGs in wastewater plays a critical role in sorption dynamics.

Sorption isotherms: The relationship between the concentration of contaminants and the sorption capacity is often described by isotherm models, such as the Langmuir and Freundlich isotherms. These models help to predict how adsorbents behave under varying concentrations.

(i) Langmuir isotherms: Suggests monolayer sorption on a homogeneous surface, where sites become saturated over time [86,87].
(ii) Freundlich isotherms: Indicates multilayer sorption on a heterogeneous surface, often observed in biochar-based adsorbents, with varying affinities for the adsorbate, demonstrating multi-layer sorption [86,87].
(iii) Competitive sorption: At high FOG concentrations, competitive sorption occurs, where molecules compete for active sites, reducing efficiency [86,87].
(iv) Concentration-dependent mechanisms: At extreme concentrations, mechanisms like coalescence or flocculation may become more dominant than sorption [86,87].

#### 2.11.3. Temperature and Viscosity

*Dynamic viscosity*: Temperature influences sorption in two significant ways. First, an increase in temperature decreases viscosity, which enhances the mobility of molecules and facilitates their diffusion toward the adsorbent. This reduction in viscosity improves contact between the adsorbate and the adsorbent surfaces, thereby promoting more effective sorption [85].

*Sorption capacity*: Numerous studies have demonstrated that the sorption capacity for fats, oils, and greases (FOGs) typically increases with temperature, particularly in physical sorption processes where enhanced kinetic energy plays a crucial role. However, excessively high temperatures can result in thermal desorption, which may diminish overall retention [84,85,86,87,88,89]. Research indicates that optimizing temperature conditions can significantly enhance sorption efficiency, especially for more viscous substances, such as engine oils, compared to lighter cooking oils [55,85,86,87,88,89,90].

#### 2.11.4. Contact Time and Sorption Kinetics

The duration of contact between the adsorbent and FOGs affects sorption efficiency. Sorption kinetics often follow either

(i) Pseudo-first-order kinetics, where sorption is diffusion-controlled;
(ii) Pseudo-second-order kinetics, where sorption is governed by chemical bonding interactions [86].

Sorption typically occurs in two phases: an initial rapid phase followed by a slower, diffusion-limited phase. The time required to reach equilibrium can vary widely based on the nature of the adsorbate and the adsorbent used. Optimizing contact time ensures complete utilization of sorption sites, improving efficiency [86].

##### Adsorbent Characteristics

The efficacy of an adsorbent depends on its surface area, porosity, functionalization, and hydrophobicity.

(i) Surface area and porosity: Materials with high surface area and well-defined pore structures enhance sorption capacity. Activated carbons, zeolites, and customized biochar are examples of effective adsorbents. Furthermore, zeolites and aerogels provide tunable surface properties, allowing enhanced hydrophobic or chemical interactions [55,85,86,87,88,89,90].
(ii) Surface chemistry: The presence of functional groups (e.g., hydroxyl, carboxyl, amine) can enhance chemical bonding interactions, increasing the strength of retention for FOGs [55,85,86,87,88,89,90].
(iii) Hydrophobicity: The hydrophobic nature of the adsorbent surface is crucial. Hydrophobic adsorbents have been found to attract and bind with non-polar OFGs efficiently [55,85,86,87,88,89,90].

#### 2.11.5. Salinity and Ionic Strength

The ionic strength or salinity of the wastewater can also influence the sorption process.

(i) Screening effect, reducing charge-based interactions: High salinity can compete with FOG molecules for sorption sites or alter the surface charge of adsorbents, modifying sorption capacity [55,85,86,87,88,89,90].
(ii) Electrostatic interactions: Changes in salinity can modify the electrostatic interactions between charged adsorbents and OFG molecules, impacting overall removal efficiency [23,55,85,86,87,88,89,90].

The mechanisms involved in the removal of oils, fats, and greases from wastewater are complex and highly dependent on a variety of factors, including pH, concentration, temperature, contact time, the characteristics of the adsorbent, and even salinity levels [5,24,38,44,51,55,81,82,83,85,89]. Understanding the interplay of these elements allows for the optimization of sorption processes and the development of more effective wastewater treatment strategies. Further research is essential to elucidate these relationships in diverse wastewater matrices and to enhance the scalability of effective treatment solutions.

### 2.12. Advanced Adsorbent Technologies: Aerogels as High-Performance Solutions

Aerogels are an emerging class of materials that offer exceptional porosity, high surface area, and hydrophobicity, making them highly effective for FOG removal and maintaining high efficiency even after multiple sorption–desorption cycles [5,24,38,44,51,55,81,82,83,85,89]. Their unique properties include the following.

#### 2.12.1. Ultra-High Surface Area and Porosity

Aerogels possess surface areas exceeding 1000 m^2^/g, maximizing available sorption sites for interaction and enhancing diffusion kinetics [5,24,38,44,51,55,81,82,83,85,89]. With porosities exceeding 90%, aerogels offer extensive void spaces, allowing for the accommodation of large volumes of oil within their structure. The interconnected nano-sized pores facilitate the diffusion of oily substances into the material [5,24,38,44,51,55,81,82,83,85,89].

#### 2.12.2. Tailored Hydrophobicity

Through chemical modifications, aerogels can be made superhydrophobic, selectively adsorbing FOGs while repelling water. This makes them ideal for applications such as oil spill cleanups and industrial wastewater treatment [5,24,38,44,51,55,81,82,83,85,89]. Surface functionalization with hydrophobic groups (such as long-chain alkyl groups) increases their affinity for non-polar substances like oils and fats, leading to effective removal from wastewater [5,24,38,44,51,55,81,82,83,85,89].

#### 2.12.3. Wettability

Hydrophobic aerogels repel water while adsorbing oils, allowing for selective removal of FOGs. This property is crucial in applications where separation of oil from water is needed [5,24,38,44,51,55,81,82,83,85,89].

#### 2.12.4. Mechanical Strength

Aerogels are extremely light but can also possess significant mechanical strength, allowing for ease of handling and application in various filtration systems. This resistance to deformation ensures the structural integrity of the adsorbent over time [5,24,38,44,51,55,81,82,83,85,89].

#### 2.12.5. Thermal Stability

Thermal stability allows robustness under varied conditions. Many aerogels demonstrate good thermal stability, making them suitable for use in various temperature conditions encountered in wastewater applications without degradation of their structure or properties [5,24,38,44,51,55,81,82,83,85,89].

#### 2.12.6. Reusability and Regeneration

Unlike conventional adsorbents, aerogels can be regenerated through thermal or solvent treatments, maintaining high efficiency across multiple sorption–desorption cycles [5,24,38,44,51,55,81,82,83,85,89].

### 2.13. Limitations and Future Perspectives of Aerogels for FOG Removal from Wastewater

#### 2.13.1. Limitations of Aerogels for FOG Removal

(a) High production costs: The synthesis of aerogels, especially silica-based and polymer-modified varieties, involves intricate and expensive processes such as supercritical drying or freeze-drying [5,24,38,44,51,55,81,82,83,85,89]. These high production costs hinder the large-scale commercial adoption of aerogels in wastewater treatment applications [5,24,38,44,51,55,81,82,83,85,89].
(b) Fragility and mechanical stability: While aerogels are celebrated for their remarkable porosity and sorption capacity, many types exhibit brittleness, rendering them susceptible to structural collapse under mechanical stress. This fragility can compromise their long-term stability and effectiveness in filtration systems [5,24,38,44,51,55,81,82,83,85,89].
(c) Limited regeneration efficiency: Although aerogels can be regenerated through thermal or solvent extraction methods, repeated regeneration cycles may result in material degradation, diminished sorption capacity, and the potential generation of secondary waste. This poses challenges for their sustainable use in wastewater treatment [5,24,38,44,51,55,81,82,83,85,89].
(d) Hydrophobicity challenges: Although hydrophobic aerogels excel at oil sorption, prolonged exposure to water and specific contaminants can alter their surface chemistry, leading to a gradual decline in sorption efficiency over time [5,24,38,44,51,55,81,82,83,85,89].
(e) Scalability issues: Transitioning from laboratory-scale aerogel fabrication to industrial-scale production presents significant hurdles. The necessity for precise control over pore structure and surface modifications complicates mass production, limiting the availability of these materials for wastewater treatment facilities [5,24,38,44,51,55,81,82,83,85,89].
(f) Selectivity concerns: The sorption efficiency of aerogels is contingent upon the types of oils, fats, and greases present in wastewater. Certain aerogels may exhibit reduced affinity for specific emulsified oils or chemically modified fats, thereby diminishing overall performance in environments with mixed contaminants [5,24,38,44,51,55,81,82,83,85,89].

#### 2.13.2. Future Perspectives for Aerogels in FOG Removal

##### Cost-Effective and Scalable Production

Future research should prioritize the development of low-cost aerogels utilizing sustainable precursors such as bio-based polymers, waste-derived silica, or hybrid materials. Innovations in ambient-pressure drying techniques could also significantly lower fabrication costs, enabling broader commercial viability.

##### Enhanced Mechanical Strength

Incorporating materials like nanofibers, graphene, or polymer reinforcements into aerogel matrices could bolster mechanical strength and resilience, enhancing durability for industrial wastewater applications.

##### Multi-Functional Aerogels

Functionalizing aerogels with catalytic or antibacterial agents could facilitate the simultaneous removal of FOGs and degradation of contaminants, thereby minimizing biofouling and enhancing long-term operational efficiency.

##### Improved Regeneration Techniques

Research aimed at developing energy-efficient and environmentally friendly regeneration methods—such as enzymatic degradation or solvent-free thermal desorption—could significantly improve the reusability and sustainability of aerogels.

##### Hybrid Sorption Systems

The integration of aerogels with other sorption or filtration technologies (e.g., membrane systems, activated carbon) could optimize overall performance, enabling more effective treatment of complex wastewater streams.

##### Smart and Responsive Aerogels

The advent of smart aerogels with stimuli-responsive properties (e.g., pH-sensitive, temperature-adaptive materials) could facilitate selective FOG sorption and controlled release, enhancing operational flexibility in wastewater treatment settings.

##### Industrial and Field Applications

Conducting large-scale pilot studies and exploring real-world applications of aerogels in wastewater treatment can provide valuable insights into their long-term stability, sorption kinetics, and cost-effectiveness across diverse operating conditions.

Currently, there is a lack of implementation of new aerogel-based technologies for water purification. To address this gap, initial steps such as the development and design of pilot-scale units, supported by pilot studies, are essential. For example, Ganesamoorthy et al. designed a pilot-scale modular system for contaminant removal from water using aerogels [17]. This modular unit consists of several horizontal stacks, each containing an optimized arrangement of aerogel-based adsorbent materials housed within a stainless-steel mesh casing. The concept’s core idea is to allow contaminated water to enter the top stack, either through pumping or gravity, and flow horizontally through the parallel aerogel columns. This configuration ensures that the water comes into contact with all the aerogel materials inside the columns before descending to the next stack. Once the aerogel reaches its maximum capacity, it can either be regenerated or replaced with inexpensive aerogels. The authors also highlighted five critical conditions that pilot-scale aerogel reactors for water purification must meet: (a) continuous operation, (b) a large surface area of aerogel in contact with water, (c) low pressure drop, (d) efficient contaminant separation, and (e) ease of aerogel regeneration.

The specialized literature includes studies examining the potential of aerogel materials, as well as other types of materials, as sorbents for contaminated water. However, with a focus strictly on this review, it can be concluded that all the research discussed regarding aerogel-based materials, such as silica-based aerogels, hybrid aerogels, and graphene oxide-based aerogels, supports their use in water remediation applications. Additionally, aerogels derived from natural polysaccharides, with cellulose being one of the most commonly used for aerogel synthesis, have also shown promise for effectively cleaning contaminated water. For most aerogels, the reusability study indicated good results after 10 sorption cycles [28,30,47,51,55,58,59,73,80]. Moreover, different studies proved that some aerogels can be used for 30 cycles [50], 35 cycles [48], 40 cycles [50], or even 50 cycles [72] with good results, as well. Based on the obtained results, the authors highlighted that the prepared aerogels, regardless of their type, can be effectively used as sorbents for the treatment of contaminated waters. An important consideration is that new types of aerogel materials can be derived or synthesized from various waste sources. This approach not only prevents waste from being discarded but also repurposes it for the production of new aerogels. In this way, principles of circular economy are upheld, contributing to environmental protection.

## 3. Conclusions

The emergence of graphene oxide-based aerogels and other next-generation aerogel materials represents a paradigm shift in oil/water separation technologies. These advanced materials not only exhibit superior sorption capacities, rapid sorption rates, and exceptional reusability but also offer a sustainable and cost-effective solution to one of the most pressing environmental challenges—fat, oil, and grease (FOG) contamination in wastewater. A particularly promising development is the utilization of waste-derived precursors, such as biomass, industrial byproducts, and recycled polymers, as raw materials for aerogel synthesis. This approach not only mitigates waste generation but also enhances the environmental and economic viability of aerogel-based water treatment solutions.

The intricate mechanisms governing the removal of oils, fats, and greases from wastewater, driven by a complex interplay of physical sorption and chemical interactions, further highlight the transformative potential of aerogels. By harnessing waste-based materials in their fabrication, researchers can optimize their structure, maximize sorption efficiency, and enhance selectivity. The intrinsic properties of aerogels—including high surface area, tunable porosity, and hydrophobicity—are further complemented by the sustainable nature of their raw materials, making them a game-changing solution for industrial and municipal wastewater management.

Looking ahead, continued innovation in aerogel engineering, particularly through surface modifications and functionalization, holds immense promise for revolutionizing pollutant removal techniques. Strategic advancements in the use of renewable and waste-derived feedstocks could significantly amplify their environmental benefits while maintaining high performance in FOG extraction and overall water purification. The future of wastewater treatment lies in the integration of these groundbreaking materials into scalable and sustainable technologies. By embracing these advancements, we can redefine wastewater management, transforming waste into valuable resources and ensuring the protection of our water systems for generations to come.

## Figures and Tables

**Table 1:** Sorption capacities for targeted oils and organic solvents.

	Targeted Contaminants	Sorption Capacity, g/g
Oils	Gasoline	55
	Diesel	72
	Pump oil	71
	Corn oil	63
	Mineral oil	83
	Motor oil	76
Organic solvents	Acetone	56
	Ethanol	52
	Toluene	65
	Hexane	45
	Chloroform	99
	DMSO	53

**Table 2:** Aerogel materials for oil/organic solvent sorption.

Aerogel Name	WCA	Porosity, %	Targeted Contaminant	Oil/Organic Solvent Sorption Capacity, g/g	Ref.
LDPE + Na_2_CO_3_	121.9°	78	diesel, biodiesel, mustard oil, ethanol, toluene, hexane	2.27–3.39	[74]
RSAMs	126°	92.6	hexane, gasoline, diesel oil, chloroform, pump oil	6.3–18.6	[75]
MFAC	142°		n-hexane, n-heptane, kerosine, toluene, xylene, diesel, crude oil, silicone oil, engine oil, chloroform	~14–42	[76]
HDCPA	148.4°	98.34	gasoline, diesel, pump oil, soybean oil, mineral oil, motor oil, acetone, cyclohexane, n-hexane, chloroform, dichloromethane, DMF, DMSO	24.4–56.5	[77]
Gelatin-based aerogel (HGAs)	131.6°	97.66	crude oil, gasoline, diesel, motor oil, vacuum pump oil, n-hexane, ethanol, carbon tetrachloride, chloroform, dichloromethane,	60.4–145.5	[78]
Stereo-complex PLA composite aerogel (SCC3)	157°	n/a	cyclohexane, CCl_4_, soybean oil, pump oil, machine oil, ethyl acetate	20.8–41.2	[79]

## Data Availability

No new data were created or analyzed in this study. Data sharing is not applicable to this article.
